# Reactive Pulmonary Capillary Hemangiomatosis and Pulmonary Veno-Occlusive Disease in a Patient with Repaired Scimitar Syndrome

**DOI:** 10.1155/2016/9384126

**Published:** 2016-03-16

**Authors:** Eva Güttinger, Bart Vrugt, Rudolf Speich, Silvia Ulrich, Fabienne Schwitz, Mattia Arrigo, Lars C. Huber

**Affiliations:** ^1^Division of Pulmonology, University Hospital Zurich and University of Zurich, 8091 Zurich, Switzerland; ^2^Institute of Surgical Pathology, University Hospital Zurich, 8091 Zurich, Switzerland; ^3^Department of Cardiology, University Hospital Zurich, 8091 Zurich, Switzerland

## Abstract

Pulmonary capillary hemangiomatosis (PCH) is a rare histological substrate within the spectrum of pulmonary arterial hypertension that possibly represents an unusual manifestation of pulmonary veno-occlusive disease (PVOD). One of the histological hallmarks of PCH is the proliferation of pulmonary capillaries in the alveolar septa that infiltrate adjacent structures such as bronchioles, vessels, and visceral pleura. The hyperplastic process involving the smallest vessels of the pulmonary vascular bed might reflect uncontrolled angiogenesis, but whether this vascular proliferation is idiopathic or, conversely, a reactive process remains to be elucidated. Here we discuss the pathogenesis of PCH exemplified by the first reported case of a young patient with repaired scimitar syndrome that developed unilateral PCH.

## 1. Background

Pulmonary capillary hemangiomatosis (PCH) and pulmonary veno-occlusive disease (PVOD) are rare types of histopathological substrates within the spectrum of pulmonary arterial hypertension (PAH). PCH is characterized by extensive proliferation of pulmonary capillaries. The etiology of these alterations is unclear. PCH has been reported to occur both idiopathically and hereditarily. In addition, PCH might represent a reactive process due to hypoxia and chronic congestion. In the context of congenital heart disease, several mechanisms including impaired pulmonary venous outflow and reactive circulatory overload might predispose to the development of PCH. Here we describe the case of a patient with scimitar syndrome that developed unilateral PCH following surgical repair. The findings discussed support the concept that PCH is a reactive angioproliferative process.

## 2. Case

We here describe a 28-year-old female patient who was diagnosed with scimitar syndrome a few days after birth. Scimitar syndrome is a very rare anomaly described to occur in about two of 100,000 births, of which females are affected in a twofold predominance. This syndrome is defined by the presence of a scimitar vein that provides an abnormal venous drainage of the right lung into the inferior vena cava. In addition, several other cardiopulmonary anomalies have been described in association with a scimitar vein (reviewed in [1]) including but not limited to hypoplasia of the right lung with consecutive dextroposition of the right heart, pulmonary sequestration, perimembranous ventricular septal defect, and a persistent ductus arteriosus. All these abnormal findings were present in the case described here.

**Figure 1:**
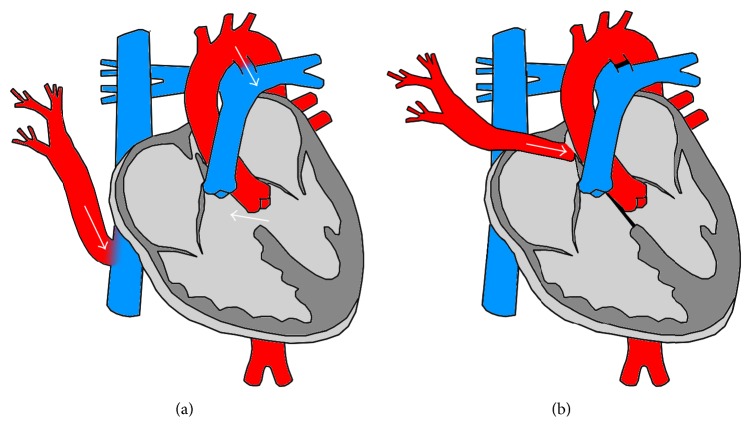
Schematic representation of preoperative situation (a) and after surgery (b). Surgical repair of the scimitar anomaly included redirection of the lung veins via patch into the left atrium, closure of the ventricular septal defect, and ligature of the ductus arteriosus.

At the age of five months, the girl underwent surgical repair to redirect the anomalous lung veins via patch into the left atrium, closure of the ventricular septal defect, and ligature of the ductus arteriosus. The pre- and postoperative situations are illustrated in Figure 1. The postoperative cardiac catheterization on the day of the operation showed complete obstruction of the right-sided pulmonary venous return and missing anterograde perfusion of the right lung. Due to the complex anatomical situation no further surgical interventions were performed and the patient remained stable with slightly impaired functional capacity for over 20 years.

At the age of 28, hemodynamic assessment by right heart catheterization confirmed complete occlusion of the right pulmonary artery and elevated pulmonary pressure (mean pulmonary arterial pressure (mPAP) of 29 mmHg with normal wedge pressure (PAWP) of 14 mmHg). Long-term oxygen therapy and PH-specific treatment with an endothelin-receptor antagonist (bosentan) were established. The course during the following years was characterized by frequent pulmonary infections but the clinical status remained stable under treatment with bosentan, diuretics, and oral anticoagulation. At the age of 34, a new episode of severe pulmonary infection with subsequent respiratory failure, septic shock, and multiorgan failure occurred. Despite maximal therapeutic efforts, the patient deteriorated further and died one day after deescalation of treatment to a palliative concept.

**Figure 2:**
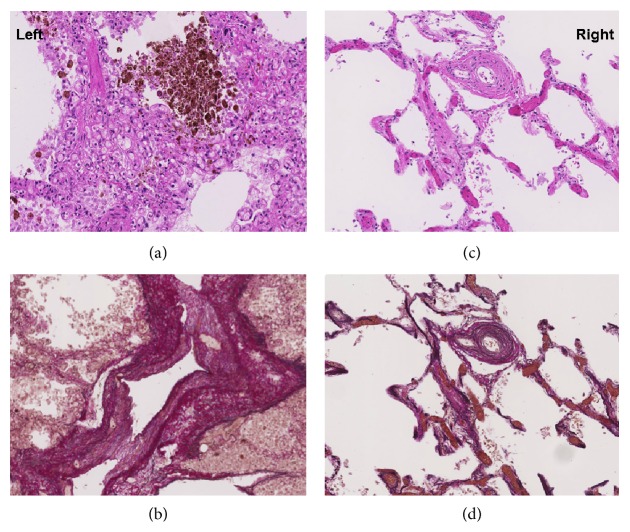
Histological pictures from the left lung (a) showing thickening of the alveolar septa due to capillary proliferation and congestion. In addition, massive iron deposition in the alveolar spaces secondary to venous obstruction is found. The Elastic van Gieson (EvG) staining (b) highlights arterialization and intimal fibrosis of venules in the interlobular septa with subtotal luminal occlusion. In contrast to the left lung, the right lung is characterized by a normal alveolar architecture with only minimal congestion (c). The EvG staining (d) shows intimal proliferation and media hyperplasia possibly related to pressure overload of the right lung but no signs of PCH or PVOD.

At autopsy, complete occlusion of the redirected right pulmonary veins and hypoplasia of the right lung were found. The lungs had dense consolidation suggesting diffuse alveolar damage as a consequence of pulmonary infection and acute respiratory distress syndrome. Massive dilatation and hypertrophy of the right ventricle was found (“cor pulmonale”) indicating severe pulmonary hypertension and right heart failure. Microscopic analysis (Figure 2) showed pulmonary veno-occlusive disease with prominent pulmonary capillary hemangiomatosis of the left lung. In particular, thickening of the alveolar septa due to capillary proliferation and congestion and massive iron deposition in the alveolar spaces secondary to venous obstruction were found (Figure 2(a)). Moreover, arterialization and intimal fibrosis of venules in the interlobular septa with subtotal luminal occlusion were described (Figure 2(b)). Conversely, the right lung was characterized by a normal alveolar architecture (Figure 2(c)). Right-sided pulmonary arteries showed alterations probably due to pressure overload with intimal proliferation and hypertrophy of the medial layer (Figure 2(d)) but no signs of PCH or PVOD, indicating a reactive, unilateral process of the left lung.

## 3. Discussion

### 3.1. Pulmonary Hypertension in Grown-Up Congenital Heart Disease (GUCH)

Pulmonary hypertension (PH) is hemodynamically defined by an increase of the mean pulmonary arterial pressure (mPAP) ≥25 mmHg and, according to the pulmonary arterial wedge pressure (PAWP), can further be distinguished in pre- and postcapillary PH [2]. PH is an umbrella term [3] and subsumes many different clinical entities, which are classified in five distinct groups shown as follows.

*Simplified Classification of Pulmonary Hypertension (Modified from [4])*

(1) Pulmonary arterial hypertension (PAH):
  (1.1) Idiopathic.
  (1.2) Heritable.
  (1.3) Drug and toxin-induced.
  (1.4) Associated with connective tissue disease, HIV infection, portal hypertension, congenital heart diseases, and schistosomiasis.
$\left(1^{\prime }\right)$ Pulmonary veno-occlusive disease (PVOD) and pulmonary capillary hemangiomatosis (PCH).
$\left(1^{\prime \prime }\right)$ Persistent pulmonary hypertension of the newborn (PPHN).
(2) Pulmonary hypertension due to left heart disease.
(3) Pulmonary hypertension due to lung disease and/or hypoxia.
(4) Chronic thromboembolic hypertension (CTEPH).
(5) Pulmonary hypertension with unclear multifactorial mechanisms.

Of note, PH related to left-sided heart disease is classified as group 2, whereas precapillary PH developing in patients with grown-up congenital heart disease (GUCH) is classified as group 1 (PAH) [4].

Pathogenic mechanisms and epidemiology of PH in the cohort of GUCH patient are ill-defined and are affected by type of the defect and type and timing of surgical repair. The association between scimitar syndrome and PH, at least in the preoperative setting, is well known [5–7]. The mainstay of therapy for scimitar syndrome is surgical repair, as performed in the case of our patient, which improves outcome. However, these patients are not cured by surgery and long-term complications including obstruction of redirected pulmonary veins as observed in the patient described here are not uncommon [8]. These patients may consecutively develop postcapillary PH. The role of PH-target therapies in this setting is of unclear benefit. Of interest, our patient developed complete occlusion of the right pulmonary artery, and elevated pulmonary pressure was measured in the contralateral (left) pulmonary artery, with normal wedge pressure. These observations implicate other pathogenic mechanisms for PH in our patient.

As such, the necroptic findings of combined PCH and pulmonary veno-occlusive disease (PVOD) in the left lung might provide a plausible explanation for this unique constellation.

### 3.2. Pulmonary Capillary Hemangiomatosis and Pulmonary Veno-Occlusive Disease

Pulmonary capillary hemangiomatosis (PCH) and pulmonary veno-occlusive disease (PVOD) represent rare, different types of histological substrates within the spectrum of PH group 1 (PAH) that involve the capillaries and the venules, respectively [9].

PCH is characterized by abnormal proliferation of capillary vessels in the pulmonary interstitium, leading to thickening of the alveolar septa. These capillaries are typically located around the bronchovascular bundles and frequently invade small pulmonary vessels. Vascular proliferation may lead to arterial or venous occlusion and nodular lesions (Figure 2). Unspecific histological hallmarks include intimal thickening and medial hypertrophy [10]. The etiology of PCH is unknown. While the onset of PCH appears to occur sporadically, two familial cases have been reported that suggest a genetic predisposition [11]. It is assumed that chronic congestion and hypoxia might play an important role in the emergence of PCH [12, 13], since angiogenesis is a commonly observed response to these triggers. In addition, inflammation and increased levels of local growth factors might be involved.

PVOD frequently appears together with PCH and is characterized by fibrous intimal thickening resulting in obstruction of the small pulmonary venules [14, 15]. Whether PCH and PVOD represent distinct entities or different phenotypical expression of the same condition with arteriocapillary or venous predomination is unclear. However, cooccurrence, as in our case here, is evident [16]. Of note, it is estimated that PCH/PVOD is found in up to 10% of patients that were primary thought to have idiopathic PAH [17].

Identification of PCH/PVOD is of major importance since treatment with PH-target vasodilators might be devastating and result in the development of pulmonary edema [18]. Anecdotal reports have described improvement of PCH/PVOD under treatment with Interferon-alpha-2a [19], but the results are not satisfying and prognosis is poor. The only curative therapy in these cases is lung transplantation [20].

While the clinical classification of PH recognizes PCH as a distinct entity [4], histological studies have provided evidence that PVOD and PCH represent the extreme ends of one disease spectrum. In addition, there is a growing body of evidence suggesting that PCH should be regarded as a reactive rather than a neoplastic angioproliferative mechanism [16]. For proliferating capillaries in the context or as a consequence of PVOD the term “secondary PCH” was presented and, conversely, “primary PCH” should be reserved for the rare idiopathic cases of PCH [16]. In congenital heart disease, however, several conditions such as capillary congestion due to impaired pulmonary venous outflow and reactive circulatory overload predispose for the development of PCH. Consistent with this hypothesis, two cases of children with PH in the context of congenital left-to-right cardiac shunts have been described in which lung biopsy showed the typical patterns of PCH [21]. Moreover, in one of these patients a congenital stenosis of the central right pulmonary arterial branch was identified. This patient developed PCH in the contralateral lung, indicating hypercirculation and volume overload as triggering factors.

The occurrence of PCH in one lung only following operative repair of scimitar syndrome complicated by unilateral pulmonary artery occlusion supports the concept that PCH is a reactive rather than a neoplastic angioproliferative process. In this context vascular shear stress induced by hypercirculation might provide a plausible explanation for the unilateral manifestation of PCH.

## Additional Points

The authors are deeply saddened by the unexpected death of their teacher and friend Rudolf Speich.

## References

[1] Gudjonsson U., Brown J. W. (2006). Scimitar syndrome. *Seminars in Thoracic and Cardiovascular Surgery. Pediatric Cardiac Surgery Annual*.

[2] Hoeper M. M., Bogaard H. J., Condliffe R. (2013). Definitions and diagnosis of pulmonary hypertension. *Journal of the American College of Cardiology*.

[3] Huber L. C., Vrugt B., Arrigo M. (2014). Pulmonary hypertension: classification and pathobiology. *Cardiovascular Medicine*.

[4] Simonneau G., Gatzoulis M. A., Adatia I. (2013). Updated clinical classification of pulmonary hypertension. *Journal of the American College of Cardiology*.

[5] Rukban H. A., Ghaihab M. A., Tamimi O., Al-Saleh S. (2014). Clinical spectrum of infantile scimitar syndrome: a tertiary center experience. *Annals of Pediatric Cardiology*.

[6] Dupuis C., Charaf L. A. C., Brevière G.-M., Abou P. (1993). ‘Infantile’ form of the scimitar syndrome with pulmonary hypertension. *The American Journal of Cardiology*.

[7] Najm H. K., Williams W. G., Coles J. G., Rebeyka I. M., Freedom R. M. (1996). Scimitar syndrome: twenty years' experience and results of repair. *The Journal of Thoracic and Cardiovascular Surgery*.

[8] Gatzoulis M. A., Webb G. D., Daubeney P. E. F. (2010). *Diagnosis and Management of Adult Congenital Heart Disease*.

[9] Langleben D. (2014). Pulmonary capillary hemangiomatosis: the puzzle takes shape. *Chest*.

[10] Lee C., Suh R. D., Krishnam M. S. (2010). Recurrent pulmonary capillary hemangiomatosis after bilateral lung transplantation. *Journal of Thoracic Imaging*.

[11] Langleben D., Heneghan J. M., Batten A. P. (1988). Familial pulmonary capillary hemangiomatosis resulting in primary pulmonary hypertension. *Annals of Internal Medicine*.

[12] Jing X., Yokoi T., Nakamura Y. (1998). Pulmonary capillary hemangiomatosis: a unique feature of congestive vasculopathy associated with hypertrophic cardiomyopathy. *Archives of Pathology and Laboratory Medicine*.

[13] Moritani S., Ichihara S., Seki Y., Kataoka M., Yokoi T. (2006). Pulmonary capillary hemangiomatosis incidentally detected in a lobectomy specimen for a metastatic colon cancer. *Pathology International*.

[14] Wagenaar S. S., Mulder J. J. S., Wagenvoort C. A., van den Bosch J. M. M. (1989). Pulmonary capillary haemangiomatosis diagnosed during life. *Histopathology*.

[15] Mandel J., Mark E., Hales C. (2000). Pulmonary veno-occlusive disease. *American Journal of Respiratory and Critical Care Medicine*.

[16] Lantuéjoul S., Sheppard M. N., Corrin B., Burke M. M., Nicholson A. G. (2006). Pulmonary veno-occlusive disease and pulmonary capillary hemangiomatosis: a clinicopathologic study of 35 cases. *The American Journal of Surgical Pathology*.

[17] Montani D., Dorfmuller P., Maitre S. (2010). Pulmonary veno-occlusive disease and pulmonary capillary hemangiomatosis. *Presse Medicale*.

[18] Humbert M., Maitre S., Capron F., Rain B., Musset D., Simonneau G. (1998). Pulmonary edema complicating continuous intravenous prostacyclin in pulmonary capillary hemangiomatosis. *American Journal of Respiratory and Critical Care Medicine*.

[19] White C. W., Sondheimer H. M., Crouch E. C., Wilson H., Fan L. L. (1989). Treatment of pulmonary hemangiomatosis with recombinant interferon alfa-2a. *The New England Journal of Medicine*.

[20] O'Keefe M. C., Post M. D. (2015). Pulmonary capillary hemangiomatosis: a rare cause of pulmonary hypertension. *Archives of Pathology & Laboratory Medicine*.

[21] Aiello V. D., Thomaz A. M., Pozzan G., Lopes A. A. (2014). Capillary hemangiomatosis like-lesions in lung biopsies from children with congenital heart defects. *Pediatric Pulmonology*.

